# Molecular-Based Score inspired on metabolic signature improves prognostic stratification for myelodysplastic syndrome

**DOI:** 10.1038/s41598-020-80918-6

**Published:** 2021-01-18

**Authors:** Juan L. Coelho-Silva, Douglas R. A. Silveira, Diego A. Pereira-Martins, Cesar A. O. Rojas, Antonio R. Lucena-Araujo, Eduardo M. Rego, João A. Machado-Neto, Israel Bendit, Vanderson Rocha, Fabiola Traina

**Affiliations:** 1 Department of Medical Imaging, Haematology, and Oncology, Ribeirão Preto Medical School, University of São Paulo, Ribeirão Preto, SP Brazil; 2 Center for Cell-Based Therapy, São Paulo Research Foundation, Ribeirão Preto, SP Brazil; 3 Hematology Division, LIM31, Faculdade de Medicina, University of São Paulo, São Paulo, SP Brazil; 4 Department of Haematology, AC Camargo Cancer Centre, Sao Paulo, Brazil; 5 Department of Internal Medicine, Ribeirão Preto Medical School, University of São Paulo, Ribeirão Preto, SP Brazil; 6 Department of Genetics, Federal University of Pernambuco, Recife, Brazil; 7 Department of Pharmacology, Institute of Biomedical Sciences, University of São Paulo, São Paulo, SP Brazil

**Keywords:** Myelodysplastic syndrome, Prognostic markers

## Abstract

Deregulated cellular energetics is formally incorporated as an emerging hallmark of cancer, however little is known about its processes in myelodysplastic syndromes (MDS). Using transcriptomic data of CD34+ cells from 159 MDS patients and 17 healthy donors, we selected 37 genes involved in cellular energetics and interrogated about its clinical and prognostic functions. Based on the low expression of *ACLY*, *ANPEP*, and *PANK1*, as well as high expression of *PKM* and *SLC25A5*, we constructed our Molecular-Based Score (MBS), that efficiently discriminated patients at three risks groups: favourable risk (n = 28; 3-year overall survival (OS): 100%); intermediate (n = 60; 76% [62–93%]) and adverse (n = 71; 35% [17–61%]). Adverse MBS risk was independently associated with inferior OS (HR = 10.1 [95% CI 1.26–81]; *P* = 0.029) in multivariable analysis using age, gender and the revised international prognostic score system as confounders. Transcriptional signature revealed that Favourable- and intermediate-risk patients presented enriched molecular programs related to mature myeloid progenitors, cell cycle progression, and oxidative phosphorylation, indicating that this cells differs in their origin, metabolic state, and cell cycle regulation, in comparison to the adverse-risk. Our study provides the first evidence that cellular energetics is transcriptionally deregulated in MDS CD34+ cells and establishes a new useful prognostic score based on the expression of five genes.

## Introduction

Myelodysplastic syndromes (MDS) are a heterogeneous group of clonal myeloid neoplasms, which are characterized by bone marrow failure, abnormal cell morphology, and increased risk for evolution to acute myeloid leukaemia^1^. The recent efforts to uncover the molecular heterogeneity of MDS, mainly by new sequencing technologies, has continually allowed the comprehensive identification of driver mutations or altered gene expression recurrently found in a recognizable fraction of patients^2,3^. Deregulated gene expression is prognostically useful in haematological neoplasms, but still underexplored in MDS^4,5^. Moreover, very few data, if any, are available considering deregulated gene expression processes of MDS-initiating cell.

Cancer cells preferentially upregulates glucose uptake and glycolysis to give rise to increased yield of intermediate glycolytic metabolites, and, as consequence, glycolysis is uncoupled from the mitochondrial tricarboxylic acid (TCA) cycle and oxidative phosphorylation (OXPHOS) in cancer cells^6,7^. This effect, also known as Warburg effect, results in reduced mitochondrial oxidative metabolism^6,8,9^, and deregulated cellular energetics is formally incorporated as an emerging hallmark of cancer^10,11^. Yet, besides the concept of how glucose metabolism influences cellular functions, studies still necessary in order to properly define if the up-regulation of anaerobic glycolysis is a true cancer cell-specific deviation or related to normal stem/progenitor cell maintenance and self-renewal mechanisms^12^.

The in-depth evaluation of MDS-initiating metabolism provided by Stevens et al. demonstrated that the CD123 + hematopoietic progenitor compartment is the clonal reservoir for MDS maintenance and evolution^13^. This CD123 + stem cells have distinctive metabolic properties, and the upregulation of protein synthesis, RNA translation, and increased oxidative phosphorylation were directly linked to MDS stem cell self-renewal and survival^13^. Mutations in the SF3B1 gene, represents a subset of MDS with favourable prognosis, results in reprogramming of mitochondrial metabolism related to decreased cellular respiration capacity in a process mediated by the mis-splicing of and downregulation of *UQCC1*^14^*.* Therefore, identification of metabolic vulnerabilities in MDS-initiating cells represents a promising strategy to better understand the pathophysiology and propose new therapeutical vulnerabilities for MDS patients.

Our rationale was to design a prognostic score interrogating the clinical and prognostic importance of transcriptionally-regulated enzymes involved in cellular energetics mechanisms of glycolysis, tricarboxylic acid cycle, and oxidative phosphorylation, and to depict the molecular process mediated by our proposed score.

## Results

### CD34+ cells from MDS show differential gene expression for cellular energetics-related genes

**Figure 1:**
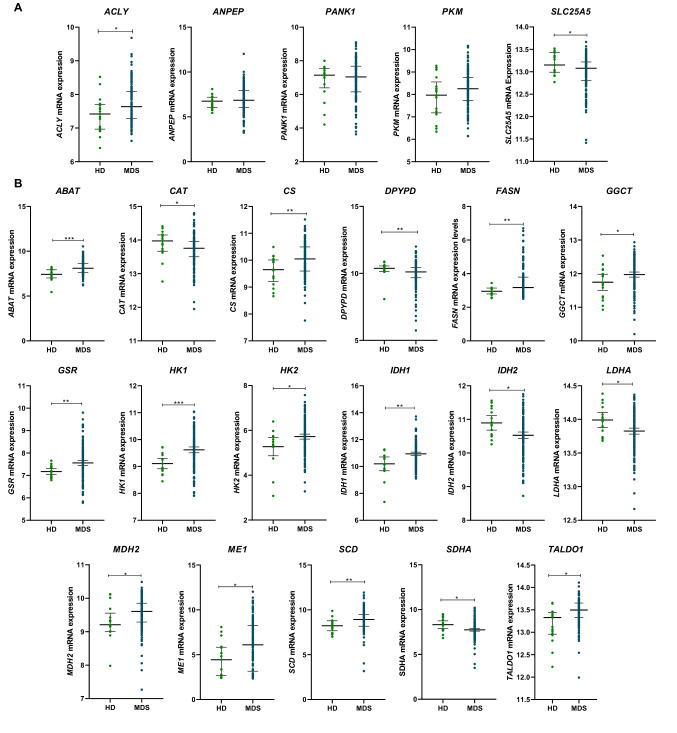
Gene expression from glycolysis and tricarboxylic acid cycle elements in CD34+ cells from healthy donors (HD) and myelodysplastic syndromes patients (MDS). A microarray-based gene expression analysis of selected genes for 17 HD and 159 MDS patients for selected genes used in Molecular Based Score (MBS) (**A**) and for genes differentially expressed between HD and MDS (**B**). Horizontal lines indicate medians and the *P* values are indicated. Notes: *P  < 0.05, **P  < 0.01, ***P < 0.001; Mann–Whitney test.

To examine the differential expression of cellular energetics-related genes, we selected 37 genes (Table 1) and normalize their expression values from microarray data for GSE58831 cohort^15^. The cohort was composed by 159 MDS patients and 17 healthy donors. Nineteen of pre-selected genes were differentially expressed between CD34+ cells from MDS patients and healthy donors (6 downregulated and 13 upregulated; Fig. 1, all *P* < 0.05).

**Table 1:** Cellular energetics-related genes selected for the study.

Cellular energetics-related genes			
*ABAT*	*GOT2*	*IDH3A*	*PFKL*
*ACLY*	*GPX1*	*IDH3B*	*PFKP*
*ANPEP*	*GSR*	*IDH3G*	*PKM*
*CAT*	*GSS*	*LDHA*	*PKMYT1*
*CS*	*GSTM1*	*LDHB*	*SCD*
*DPYPD*	*HK1*	*MDH2*	*SDHA*
*ERCC2*	*HK2*	*ME1*	*SLC2A5*
*FASN*	*IDH1*	*OGDH*	*SLC25A5*
*GAD1*	*IDH2*	*PANK1*	*TALDO1*
*GGCT*

### Molecular-Based Score efficiently discriminates MDS patients at differential risk and is associated with clinical and molecular characteristics

To interrogate the prognostic capacity for each selected gene, we dichotomized the gene expression in high- or low-expression according to their receiving operating characteristics (ROC) curve and the C-index. Fifteen genes were associated with prognosis in a univariate analysis, while multivariate analyses identified expression of 5 genes as independent prognostic factors: *ACLY* (HR: 0.48; 95% CI 0.24–0.96; *P* = 0.04), *ANPEP* (HR: 2.16; 95% CI 1.08–4.31; *P* = 0.02), *PANK1* (HR: 0.43; 95% CI 0.19–0.98; *P* = 0.04), *PKM* (HR: 2.01; 95% CI 1.02–3.93; *P* = 0.04), and *SLC25A5* (HR: 0.49; 95% CI 0.27–0.99; *P* = 0.05) (Table 2). The molecular-Based Score (MBS) was calculated by summing 1 for every gene as a risk factor. The MBS varied from 0 to 5 and was stratified as: MBS Favourable-Risk = 0 (MBS-FR; 18% [28/159]); MBS Intermediate-Risk = 1 (MBS-IR; 38% [60/159]) and Adverse-Risk: ≥ 2 (MBS-AR; 44% [71/159]).

**Table 2:** Genes associated with overall survival in Cox Proportional Hazard Model.

Target	Univariate analysis			Multivariate analysis		
	Hazard Ratio^1^	95%CI	*P*	Hazard Ratio^1^	95%CI	*P*
*GAD1*	0.34	0.17–0.66	0.001	0.73	0.3–1.73	0.47
***ANPEP***	2.73	1.44–5.17	0.002	2.16	1.08–4.31	0.02
***ACLY***	0.38	0.21–0.72	0.002	0.48	0.24–0.96	0.02
*DPYPD*	3.19	1.33–7.65	0.008	2.22	0.9–5.48	0.08
*MDH2*	0.43	0.23–0.81	0.009	0.6	0.31–1.21	0.15
***SLC25A5***	0.44	0.24–0.83	0.01	0.49	0.27–0.99	0.04
*GOT2*	0.37	0.17–0.80	0.01	0.58	0.24–1.36	0.21
***PKM***	2.24	1.18–4.23	0.01	2.01	1.02–3.93	0.04
*SLC2A5*	2.17	1.16–4.05	0.01	1.84	0.88–3.87	0.1
*GSS*	0.46	0.24–0.86	0.01	0.65	0.32–1.31	0.22
*LDHB*	0.42	0.21–0.85	0.01	0.61	0.28–1.28	0.19
***PANK1***	0.42	0.21–0.86	0.01	0.43	0.19–0.98	0.04
*IDH3G*	0.25	0.07–0.84	0.02	0.34	0.11–1.16	0.08
*SCD*	2.31	1.1–4.87	0.02	1.56	0.72–3.38	0.25
*SDHA*	0.42	0.18–0.98	0.04	0.51	0.21–1.17	0.11

*IC95%* confidence interval of 95%.

Genes highlighted in bold were independently associated with overall survival and selected to Molecular Based Score.

^1^Hazard ratios (HRs) > 1 or < 1 indicate that higher or lower gene expression predicts increased risk of death, respectively.

**Figure 2:**
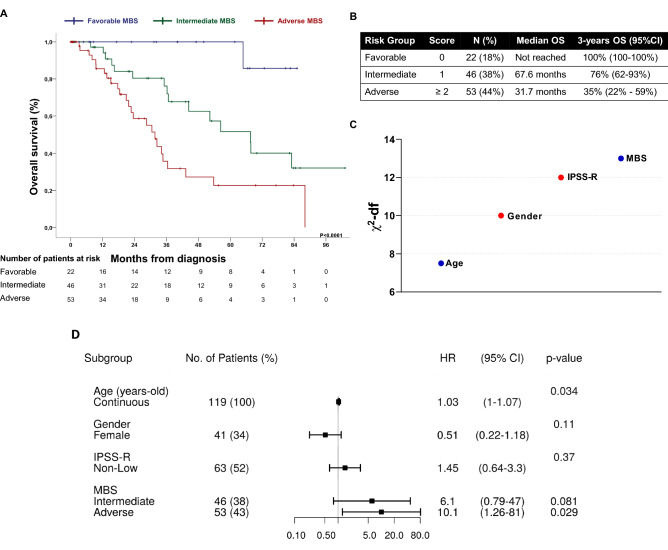
Survival analyses of Molecular-Based Score (MBS) on overall survival (OS) of myelodysplastic syndrome. (**A**) Kaplan–Meier curves for the three MBS risk categories. (**B**) MBS was built based on gene expression of *ACLY*, *ANPEP*, *PANK1*, *PKM* and *SLC25A5.* MBS efficiently identify three risk groups. (**C**) Significance (χ^2^-statistic) of each covariate for prediction of OS in the multivariate model, in which higher values represents increased predictive capacity; df: degrees of freedom. (**D**) Forest plot for multivariable analysis identified adverse risk-MBS and age as independent predictors of OS. Hazard ratios (HR) > 1 indicates that increasing values for continuous variable or the first factor for categorical variables has the poorer outcome. HR and their respective 95% confidence interval (95%CI) are indicated with black square and a line, respectively. IPSS-R non-low patients included intermediate, high and very-high patients.

Molecular-Based Score efficiently discriminated patients at different risks groups: MBS-FR (3-year overall survival (OS): 100%; median time [MT]: not reached); MBS-IR (3-year OS: 76% [95% CI 62–93%]; MT: 67.6 months [95% CI 48.3–86.8]) and MBS-AR (3-year OS: 35% [95% CI 17–61%]; MT: 31.7 months [95% CI 21.2–42.1]) (Fig. 2A,B). The univariate HRs for IR *versus* FR and AR *versus* IR were 8.99 (95% CI 1.19–68.1; *P* = 0.02) and 20.1 (95% CI 0.2.71–149; *P* = 0.003), respectively (Supplemental Fig. 1). After multivariate adjust, MBS-AR was the most significant covariate as measured by the Wald chi-square statistic and was independently associated to inferior OS (HR = 10.1 [95% CI 1.26–81]; *P* = 0.029) (Fig. 2C,D). We also identified increased age as an independent prognostic covariate in our model (HR = 1.03 [95% CI 1–1.87]; *P* = 0.034), representing an increment of 3% of risk of death by year of age at diagnosis (Fig. 2D).

**Figure 3:**
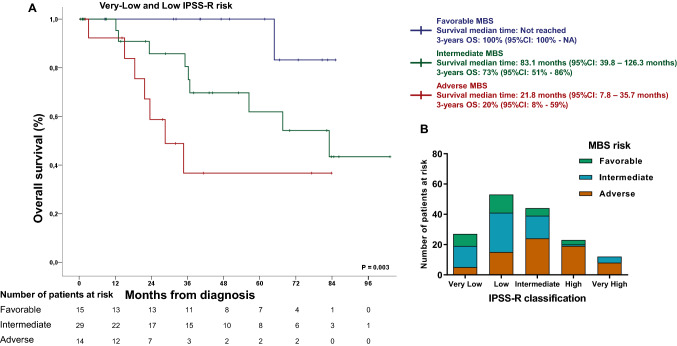
Molecular-Based Score (MBS) prognostic prediction in IPSS-R very-low- and low-risk patients, and distribution across all IPSS-R risk categories. (**A**) Kaplan–Meier curves of MBS on overall survival (OS) of IPSS-R very-low and low-risk myelodysplastic syndrome. (**B**) Distribution of MBS across all IPSS-R classification.

Patients classified as adverse by MBS had significantly decreased platelets counts (median for FR:250 × 10^3^/µL; IR: 157 × 10^3^/µL and AR: 109 × 10^3^/µL; *P* = 0.001) and absolute neutrophil counts (FR:2.5 × 10^3^/µL; IR: 2.3 × 10^3^/µL and AR: 1.3 × 10^3^/µL; *P* = 0.003), while presented higher percentages of bone marrow blasts (FR: 2.5%; IR: 3% and AR: 8.5%; *P* < 0.001). MBS risk categories were differently distributed across World Health Organization (WHO) MDS entities and IPSS-R classification (both *P* < 0.001). According to recurrently mutated genes, MBS-AR showed lower frequency of mutations in *SF3B1* (FR:50%; IR: 32% and AR: 15%; *P* < 0.001), and higher frequency of mutations in *RUNX1* (FR: 0; IR: 2% and AR: 13%; *P* = 0.03) (Table 3). Collectively, these data suggest a link between MBS and pathophysiology of MDS. MBS Receiving-operating characteristics concordance statistic (ROC C-statistic) was 0.70 (95% CI 0.62–0.78; Table 4), representing a 20% improvement in OS prediction when compared with IPSS-R (Δ-AUC, 0.13; 95%CI 0.02–0.22; *P* = 0.01). According to IPSS-R risk stratification, MBS retained its prognostic prediction function when analysed in IPSS-R very-low- and low-risk patients (Fig. 3A) and was widely distributed across all risk categories (Fig. 3B). For non-low IPSS-R patients (i.e., intermediate, high, and very-high), MBS-favourable patients presented a distinctive superior outcome (Supplemental Fig. 3). Of note, none of favourable MBS patients classified as non-low IPSS-R deceased, while 4 of 6 low risk IPSS-R classified as adverse by MBS died with median survival of 18.4 months (Supplemental Table 3).

**Table 3:** Baseline characteristics of patients included for Molecular-Based Score.

Characteristics	All patients			Molecular-Based Score									
				Favorable risk			Intermediate risk			Adverse risk			
	No	%	Median (range)	No	%	Median (range)	No	%	Median (range)	No	%	Median (range)	*P* value
N	159	100		28	17.6		60	37.8		71	44,6		
**Gender**													0.08
Female	57	35.8		15	46.4		21	35		21	30		
Male	102	64.2		13	53.6		39	65		50	70		
Age, years			67 (19—87)			63 (32—82)			67 (19–87)			67 (33–87)	0.439
Bone marrow blasts, %			4 (0—63)			2.5 (0–14)			3 (0–63)			8.5 (0–46)	< 0.001
Hemoglobin, g/dL			9.5 (4.5–14.6)			10 (6.9–11.9)			9.45 (5.4–14.6)			9.8 (4.5–14.4)	0.952
Absolute neutrophil count, × 10^3^/µL			1.8 (0.08–15.2)			2.5 (0.91–5.36)			2.3 (0.38–6.4)			1.3 (0.08–15.2)	0.003
Platelets, × 10^3^/µL			152 (10–1042)			250 (38–787)			157 (16–604)			109 (10–1042)	0.007
**Transfusion dependency**													0.627
Yes	58	44.3		9	57.1		27	55.1		30	52.7		
No	73	55.7		16	32.1		22	44.9		27	47.3		
**IPSS-R**													< 0.001
Very-low	27	17.0		8	28.6		14	23.3		5	7.1		
Low	53	33.3		12	42.9		26	43.3		15	21.4		
Intermediate	44	27.7		5	17.9		15	25		24	33.8		
High	23	14.5		3	10.7		1	1.7		19	26.8		
Very-high	12	7.5		0	0		4	6.7		8	11.3		
**WHO 2008 category**													< 0.001
RA	13	8.2		1	3.6		9	15		3	4.2		
RCMD	27	17.0		1	3.6		16	26.7		10	14.1		
RCMD-RS	22	13.8		7	25		10	16.7		5	7		
RARS	14	8.8		8	28.6		4	6.7		2	2.9		
RARS-T	6	3.8		4	14.3		2	3.3		0	0		
MDS with 5q-	6	3.8		2	7.1		4	6.7		0	0		
RAEB	28	17.6		1	3.6		9	15		18	25.3		
RAEB-2	28	17.6		4	14.3		3	5		21	29.6		
AML-MDS	7	4.4		0	0		1	1.7		6	8.4		
NA	8	5.0		0	0		2	3.3		6	8.4		
Mutations													
*SF3B1*	37	29.8		14	50		15	31.9		8	14.8		< 0.001
*TET2*	33	20.8		5	21.7		14	29.8		14	25.6		0.765
*ASXL1*	21	13.2		2	8.7		6	12.8		13	24.1		0.161
*SRSF2*	16	10.1		1	4.3		6	12.7		9	16.7		0.336
*DNMT3A*	13	8.2		3	13		7	14.9		3	5.5		0.282
*RUNX1*	8	6.5		0	0		1	2.1		7	12.9		0.03
*U2AF1*	8	6.5		1	4.3		1	2.1		6	11.2		0.168

IPSS-R: Revised International Prognostic Score System**;** RA: refractory anemia; RCMD: refractory cytopenia with multilineage dysplasia; RCMD-RS: refractory cytopenia with multilineage dysplasia with ring sideroblasts; RARS: refractory anemia with ring sideroblasts; RARS-T: refractory anemia with ring sideroblasts and thrombocytosis; RAEB: refractory anemia with excess blasts. AML-MDS: acute myeloid leukemia with myelodysplastic alterations; NA: Not available.

**Table 4:** Overall survival for IPSS-R and molecular based score (MBS).

Factors	2-years OS (95%CI)	3-years OS (95%CI)	*P* value^1^	AUC
**IPSS-R**				
Very-low	78% (59–100%)	78% (59–100%)	0.004	0.57 (0.46–0.67)
Low	85% (73–98%)	74% (60–92%)		
Intermediate	69% (50–93%)	43% (23–80%)		
High	69% (44–100%)	55% (30–100%)		
Very-high	43% (18–100%)	43% (18–100%)		
**MBS**				
Favourable	100% (100–100%)	100% (100–100%)	< 0.001	0.70 (0.62–0.78)
Intermediate	80% (67–96%)	76% (62–93%)		
Adverse	59% (44–77%)	35% (22–59%)		

*IC95%* confidence interval of 95%; *IPSS-R* International Prognostic Score System-Revised.

^1^Log-rank test.

### Internal validation

Based on the unique characteristics of this cohort, mainly by microarray-based transcriptomic data from CD34+ cells, we decided to internally validate our data using the bootstrap resampling procedure. The bootstrap results are depicted in Table 5, and, for all time-points, the procedure yielded a mean 95%CI virtually identical to its original match. In addition, the pairwise hypothesis test showed a strong significance (*P* < 0.001) for the difference across the distributions’ means for all comparisons. The procedure showed the stability of MBS prediction for 2- and 3-years OS and reinforce the validity of its prediction in a new, but similar, patient collective.

**Table 5:** Bootstrap (R = 1000) for 2-years and 3-years OS.

MBS	2-years OS (95%CI)	*P* value^1^	3-years OS (95%CI)	*P* value^1^
Favourable	100% (100–100%)	< 0.001	100% (100–100%)	< 0.001
Intermediate	81% (62–93%)		76% (57–90%)	
Adverse	58% (42–75%)		35% (19–56%)	

^1^Kruskal-Wallis Test.

### Molecular-Based Score categories are associated with differential gene expression signatures

**Figure 4:**
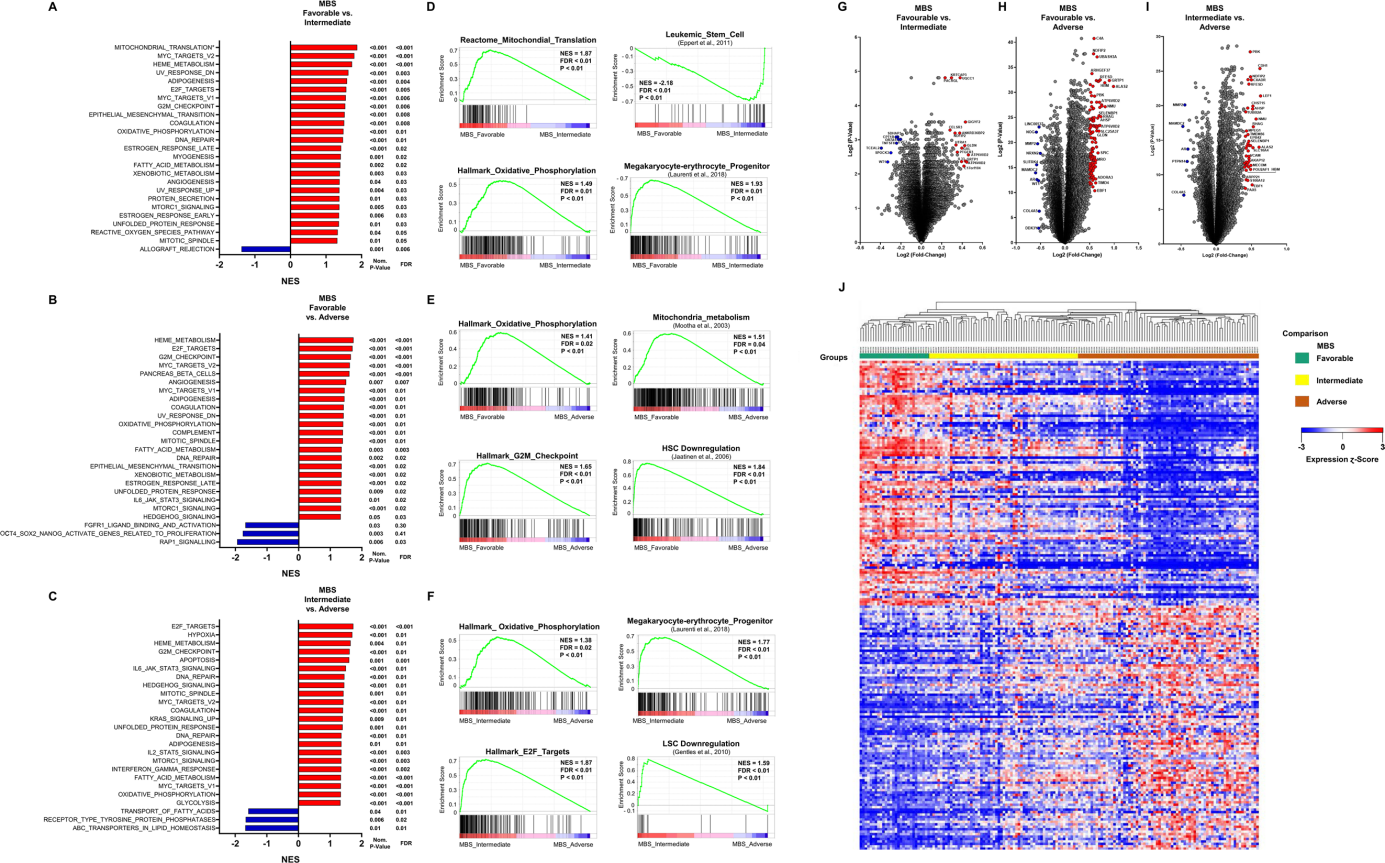
Molecular-Based Score (MBS) entities are associated with differential transcriptomic programs. (**A**–**C**) Gene set enrichment analysis (GSEA) with compiled modules from Hallmarks of the molecular signatures database. * indicates a GSEA for Reactome database. Specific comparisons are indicated in the figure. False discovery rate (FDR) < 0.25, normalized enrichment score (NES) >|1.5|. (**D**–**F**) Representative enrichment graphs from ranked GSEA analysis. Specific comparisons are indicated in the figure. (**G**–**I**) Volcano plots depicting the extent (x-axis) and significance (y-axis) of differential gene expression for each gene comparing favourable versus (vs) intermediate, favourable vs adverse, and intermediate vs adverse MBS categories, respectively. (**J**) Heat map summarizing expression of the top 200 differentially expressed genes across MBS entities. Colour intensity represents the by ɀ-score within each row. Expression values for genes represented by multiple probes reflect the median across-array intensity and the gene expression profiles were clustered using the K-means algorithm. Heat map was constructed using Morpheus (https://software.broadinstitute.org/morpheus).

To further understand the potential mechanisms by which MBS entities regulate hematopoietic progenitor-associated transcriptional programs, we comprehensively compared the transcriptomics signatures among MBS risk categories. Gene set enrichment analysis (GSEA) revealed that increasing MBS risk (i.e. favourable *versus* (*vs*) intermediate; favourable *vs* adverse; and intermediate *vs* adverse) was consistently characterized by upregulation of genes related to oxidative phosphorylation, upregulation of controllers circuits of the cell cycle progression (e.g. G2M_checkpoint and E2F_Targets), and fatty-acid metabolism (Fig. 4A–C; Supplemental Table 1). For specific comparisons, favourable MBS patients were positively enriched with a transcriptional program of megakaryocytic-erythroid progenitor (MEP)^16^ and negative enrichment with leukemic stem cell signature^17^ compared with adverse patients (Fig. 4D). In accordance with the previous observations, favourable patients presented a positive enrichment with mitochondria metabolism^18^ and downregulated genes in hematopoietic stem cell^19^ (Fig. 4E). Adverse MBS patients presented negative enrichment with MEP and downregulated genes in leukemic stem cell (Fig. 4F).

Applying stringent statistical criteria (upregulation: log2 fold change > 1.5; downregulation < -1.5, all *P* < 0.05), we identified differentially expressed genes (DEG) for the following comparisons: favourable vs intermediate (8 upregulated and 16 downregulated), favourable vs adverse (10 upregulated and 129 downregulated) and intermediate vs adverse (5 upregulated and 42 downregulated) (Fig. 4G-I). Unsupervised hierarchical clustering of transcriptomic data clearly segregated favourable and adverse patients with distinctive DEG signature (Fig. 4J). Taken together, these results suggest that MBS risk categories can efficiently stratify differential transcriptional programs, especially related to cellular energetics and hematopoietic progenitor differentiation.

## Discussion

Here, we described a new prognostic scoring system for patients with MDS based on gene-expression of five metabolic enzymes in CD34+ cells, useful to distinguish patients at three risk categories. Regardless of the wide clinical application of IPSS-R^20^ for risk assessment in MDS, refining its prognostic function with additional clinical information^21^, flow-cytometry^22^, or mutations^23^ has been of great interest, whereas gene expression analysis it has been underexplored for this purpose. Our proposed MBS efficiently discriminate very-low and low IPSS-R in three risk categories, as well as identified a subset of very favourable prognosis among non-low IPSS-R patients. As far as we know, only two gene expression-based risk scores were published for MDS patients^4,5^, and because both of them have used bulk of bone marrow mononuclear cells, its translation to MDS biology is limited. We have demonstrated that deregulated gene expression in at least two of selected genes is capable to independently predict poorer OS in MDS, with superior prediction capacity than IPSS-R.

The high degree of molecular complexity in MDS represents a challenge to properly define the contribution of all alterations to the pathophysiology of these diseases. Moreover, the majority of MDS biomarkers is still based on mutational profiling^24,25^. Despite the limitation in implementing molecular investigations in clinical setting, particularly in low- and middle-income countries, several initiatives had efficiently established molecular tests validated for risk assessment for other myeloid neoplasms^26,27^.

The strong prognostic function of the MBS across the spectrum of MDS entities and risk categories indicates that perturbations caused by driver molecular alterations might result in metabolic reprogramming and that the MBS is capable to efficiently capture these downstream consequences. Based on Molecular-Based Score classification, we were able to identify patients with differential transcriptional programs that reflect an increased mitochondrial respiration capacity, protein synthesis and, molecular signature related to more mature hematopoietic progenitors in MBS favourable- and intermediate-risk comparing with adverse-risk. Stemness-related transcriptional signature is recognized as a relevant predictor of inferior survival in acute myeloid leukaemia^26^. Moreover, more mature hematopoietic progenitors, such as multipotent and myeloid progenitors, show increased baseline oxygen consumption, mitochondrial ATP production, and respiratory capacity than HSC^28^. Therefore, is conceivable that high MBS risk patients have CD34^+^ cells in a more undifferentiated state, related to its reduced mitochondrial respiration capacity and cell cycle progression. As a consequence, this delayed haematopoiesis could result in more severe cytopenia in peripheral blood and accumulation of blasts in the bone marrow.

Using advanced stage MDS patients, it has already been demonstrated that CD34^+^CD123^+^ primitive stem cell is responsible for clonal maintenance and expansion. This compartment has distinctive metabolic characteristics, with activation of protein synthesis machinery and increased oxidative phosphorylation, in comparison to CD34^+^CD123^−^ counterparts^13^. Conversely, in our study, we demonstrated that lower MBS risk was associated with increased oxidative phosphorylation and protein biosynthesis signatures. We may hypothesize that metabolic reprogramming in CD123^+^ cells occurs to a different extent for non-advanced stage MDS patients. Indeed, the *IL3RA* is not differentially expressed among MBS risk categories (Supplemental Fig. 2). As we used transcriptomic from CD34+ bulk cells, the molecular signatures that we observed are probably related to other more frequent subsets of cells. In addition, ectopic expression of SF3B1 mutations in breast cells was associated with disrupted mitochondrial respiration capacity^14^. SF3B1 mutated MDS is considered as having a good prognosis and was recently proposed as a specific disease subtype^29^. Favourable MBS-risk was associated with SF3B1 mutation (Table 3) and as having an oxidative phosphorylation signature. Then, we propose that disruption of mitochondrial complex III mediated by mutant SF3B1 could be dependent on the cellular context, and the metabolic consequences of SF3B1 mutations in CD34^+^ of MDS patients still of major importance.

Ideally, validation of a new prognostic model should determine its capacity in a new data-set scenario. However, external validation is not feasible in most situations. The cohort used in this manuscript shows some unique characteristics, such as: 1) transcriptomic data from microarray of CD34^+^ cells, 2) and availability of clinical and demographic data, such as survival, gender, haematological parameters and risk stratification, as well as mutation data. To overcome the impossibility of external validation, we considered internal validation using bootstrap resampling method to evaluate both predictive accuracy and to check overfitting. Of note, this procedure is aligned with the best analytical rigor and was widely used in clinical studies with singular characteristics^30–33^. Independent external cohorts’ validations and evaluations in the context of response to different therapies would reinforce the clinical relevance of the proposed score.

The proposition of more efficient and less toxic new therapies is dependent on the ability to exploit a specific weakness that is inherited preferentially in the neoplastic stem cell population. The identification of the MBS for MDS patients contributes to the knowledge of disease pathobiology and provides novelty data according to altered cellular metabolism of the MDS-initiating cell.

## Methods

### Clinical and molecular data

Patients’ features, mutational status and CD34^+^ cells transcriptome data from 159 MDS patients and 17 healthy donors are publicly available at *Gene Expression Omnibus* (GEO-NCBI; GSE58831)^34^. Briefly, classification of MDS was updated at sample collection and made according to World Health Organization criteria^35^, while risk stratification determined by IPSS-R^20^. All patients and healthy controls were from Europe and the centres included: Oxford and Bournemouth (UK), Duisburg (Germany), Stockholm (Sweden) and Pavia (Italy). Baseline features for entire cohort are included in Table 3.

Expression of 37 genes that codify to enzymes related to glycolysis, mitochondrial tricarboxylic acid cycle and oxidative phosphorylation transcriptionally regulated and previously listed as a phenotypic modifiers across different cancer types^36–38^ were selected to interrogate its differential gene expression and predictive outcome function (Table 1).

### Transcriptomic analysis

Diagnosis CD34^+^ cells were enriched from mononuclear cells using CD34 MicroBeads (Miltenyi Biotec, Germany). For each sample, total RNA was extracted using TRIZOL (Invitrogen, UK) and 50 ng were amplified and labelled using Two-Cycle cDNA Synthesis and the Two-Cycle Target Labelling and Control Reagent kits (Affymetrix, USA). Ten µg of cRNA was hybridized to Affymetrix GeneChip Human Genome U133 Plus 2.0 arrays (Affymetrix, USA), covering 47 000 transcripts. Normalized gene expression was calculated using a multichip analysis approach^39^. Mutation data were obtained by targeted gene sequencing, using Illumina Platform, designed to cover 111 genes implicated in myeloid neoplasms pathobiology^40^.

The quantile normalized gene expression was used for a ranking using limma-voom package at Galaxy (https://usegalaxy.org/) comparing MBS groups (i.e. favourable versus intermediate, favourable versus adverse, and intermediate versus adverse). Pre ranked gene set enrichment analysis (GSEA) was performed using GSEA 4.0.3 software^41^. The gene sets curated by MSigDB hallmark, reactome, hematopoietic progenitors, mitochondrial, and apoptosis were selected for comparisons. Volcano plots computing differentially expressed across MBS entities were constructed correlating the Log_2_-adjusted *P* value and Log_2_-Fold-Change in GraphPad Prism 8.0 (GraphPad Software, USA). Heat map was constructed to represent top differentially expressed genes in MBS risk groups using the online available tool Morpheus (https://software.broadinstitute.org/morpheus).

### Statistical considerations

Descriptive analyses were performed for patient baseline features. Fisher’s exact test or Chi-square test, as appropriate, was used to compare categorical variables. Non-parametric Mann–Whitney test was used to compare continuous variables.

In order to optimize the cut off selection for gene expression, we opted to use “cutpointr” package and automatically determined the critical points for each 37 genes using receiver operating characteristic curve analysis^42^ and the C-index^43^ pre-selected for our score (Table 1). After dichotomization, we evaluated the predictive capacity of each gene (Table 2) in a univariate and multivariate way by Proportional Hazard Cox regression analysis using the “Cox_HR” function of “SurvivalAnalysis” package^44,45^. Genes (n = 11) significantly associated with survival in univariate analysis were individually considered in multivariate analysis using age, gender, and IPSS-R stratification as cofounders. Five genes independently predicted OS and were selected for MBS estimation.

MBS was calculated by computing 1 for every molecular risk factor, e.g. high expression of *ANPEP* and *PKM*, and low expression of *ACLY*, *PANK1* and *SLC25A5*, varying from 0 (summing zero molecular risk factor) to 5 (summing all five molecular risk factor). MBS risk groups were determined by Kaplan-Meyer inspection^46^, and were defined as MBS-Favourable for patients without molecular risk factor, MBS-Intermediate for patients with one molecular risk factor and as MBS-Adverse with two or more molecular risk factors.

To determine the predictive capacity for MBS, a receiver operating characteristic (ROC) curve and the respective concordance statistics (C-statistics) were performed. The respective area under the curve (AUC) were derived from an R implementation of DeLong’s algorithm^47^. To determine if MBS predictive capacity is superior to IPSS-R, we calculated differences between AUC (Δ-AUC) as Δ-AUC = AUC_MBS_ − AUC_IPSS-R_. For this purpose, we performed 10,000 bootstrap resampling procedure and calculated the Δ-AUC for each interaction. Positive values represent that MBS performed better than IPSS-R^48^.

The bootstrap resampling procedure performed 1,000 resampling of the original cohort and calculated all clinical endpoints in two different time points (2-year, and 3-year) for three MBS-categories (favourable-, intermediate- and adverse-risk MBS). The procedure also estimated their respective 95% confidence interval (CI) computing the bias-corrected and accelerated bootstrap interval.

Proportional hazards (PH) assumption for each continuous variable of interest was tested. Linearity assumption for all continuous variables was examined in logistic and PH models using restricted cubic spline estimates of the relationship between the continuous variable and log relative hazard/risk. All *P* values were two sided with a significance level of 0.05. All calculations were performed using Stata Statistic/Data Analysis version 12 (Stata Corporation, USA), Statistical Package for Social Sciences 19 (SPSS 19) and R 3.5.2 (The CRAN project, www.r-project.org) software.

## Supplementary information

Supplementary Information 1.

Supplementary Information 2.

Supplementary Information 3.

Supplementary Information 4.
