# Supplementary material for: Molecular-Based Score inspired on metabolic signature improves prognostic stratification for myelodysplastic syndrome
Source: Sci Rep. 2021 Jan 18;11:1675. doi: 10.1038/s41598-020-80918-6 (PMC7814118; doi:10.1038/s41598-020-80918-6)
Supplement: Supplementary file 1 — Supplementary Information 1. [file 41598_2020_80918_MOESM1_ESM.pdf]

MYELOID NEOPLASIA

Molecular-Based Score inspired on metabolic signature improves prognostic stratification for Myelodysplastic Syndrome

Juan L Coelho-Silva, [1,2] Douglas R A Silveira, [3] Diego A Pereira-Martins, [2,4], Cesar A O Rojas, [2] Antonio R Lucena-Araujo, [5] Eduardo M Rego, [2,3] João A Machado-Neto, [6] Israel Bendit, [3] Vanderson G Rocha, [3] Fabiola Traina[1,2*]

Affiliations: [1]Departament of Medical Imaging, Haematology, and Oncology, Ribeirao Preto Medical School, University of Sao Paulo, Ribeirao Preto, SP, Brazil; [2]Center for Cell-Based Therapy, Sao Paulo Research Foundation, Ribeirao Preto, SP, Brazil; [3]Hematology Division, LIM31, Faculdade de Medicina, University of Sao Paulo, Sao Paulo, SP, Brazil; [4]Department of Internal Medicine, Ribeirao Preto Medical School, University of Sao Paulo, Ribeirao Preto, SP, Brazil; [5]Department of Genetics, Federal University of Pernambuco, Recife, Brazil; [6]Department of Pharmacology, Institute of Biomedical Sciences, University of Sao Paulo, Sao Paulo, SP, Brazil

Supplementary figure 1: Univariate survival analyzes by Cox Proportional Hazard Model. Forest plot with covariates used for univariate analysis identified age, gender, international prognostic score system-revised (IPSS-R) classification and intermediate- and adverse-risk molecular-based score (MBS) as predictors of overall survival. Hazard ratios and 95% confidence interval (95%CI) are indicated with black square and a line, respectively. Non-low patients included intermediate, poor and very-poor IPSS-R patients.

Supplementary figure 2: *IL3RA* expression according to Molecular-Based Score (MBS) risk classification. A microarray-based gene expression analysis of CD123 gene (*IL3RA*) in myelodysplastic syndromes (MDS) Horizontal lines indicate medians and interquartile range. P-value is indicated. Kruskall-Wallis test.

Supplementary figure 3: Molecular-Based Score (MBS) prognostic prediction in IPSS-R intermediate, high, and very-high-risk patients. Kaplan-Meier curves of MBS on overall survival (OS) of IPSS-R intermediate, high, and very-high-risk myelodysplastic syndrome.

Supplementary table 1: List of ranked gene expression comparing Molecular-Based Score (MBS) risk entities transcriptomics.

Supplementary table 2: List Gene sets enrichment analysis (GSEA) comparing Molecular-Based Score (MBS) risk entities molecular signatures.

Supplementary table 3: Overall survival and patient distribution according to the International Prognostic Score System-Revised (IPSS-R) and Molecular-Based Score (MBS).

Supplementary figure 1

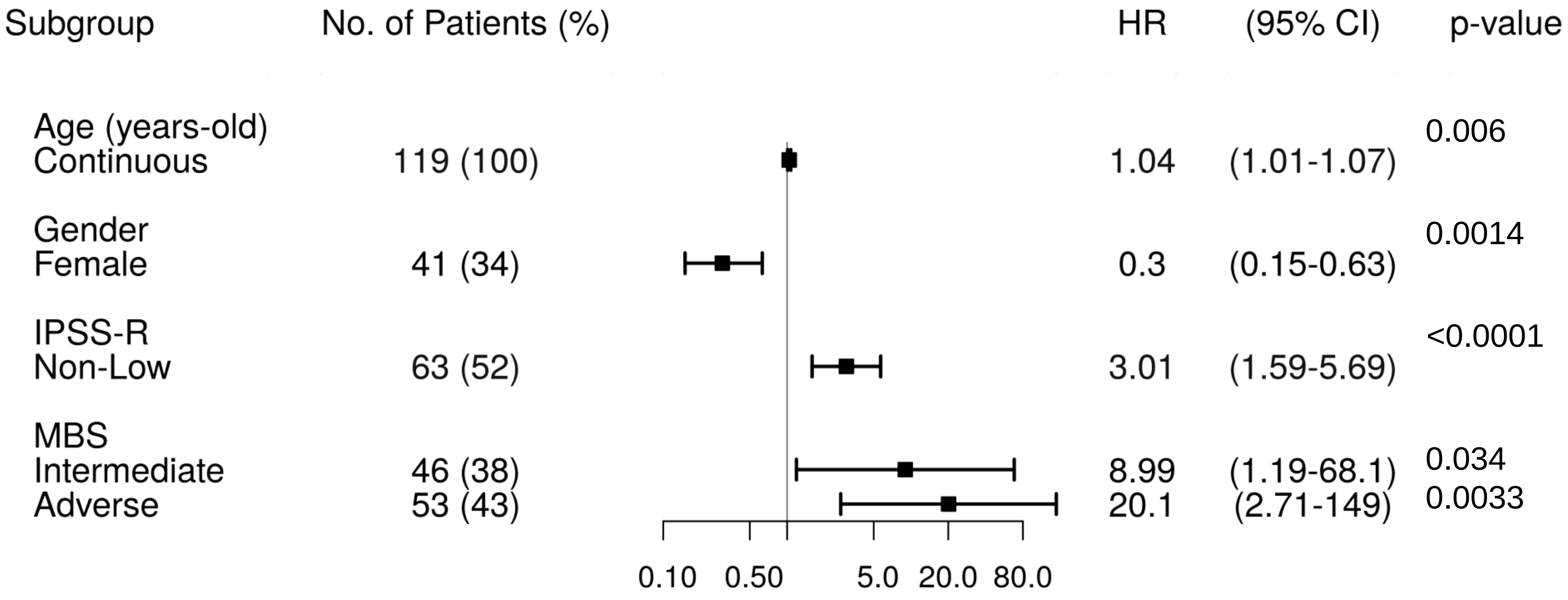

Supplementary figure 2

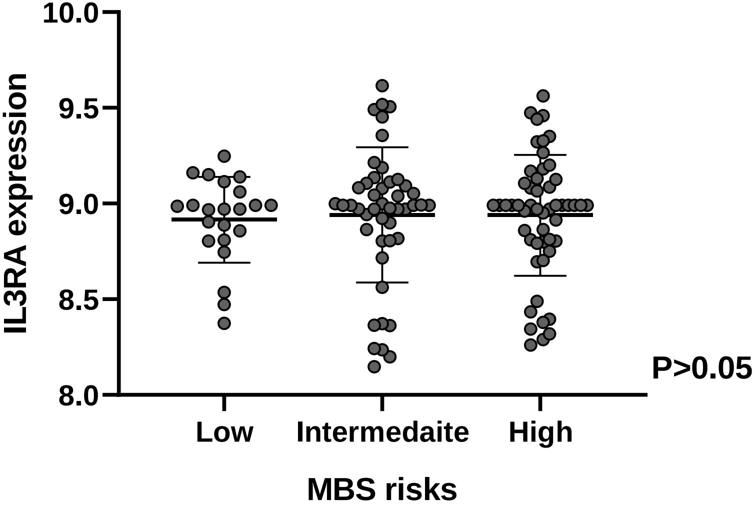

Supplementary figure 3

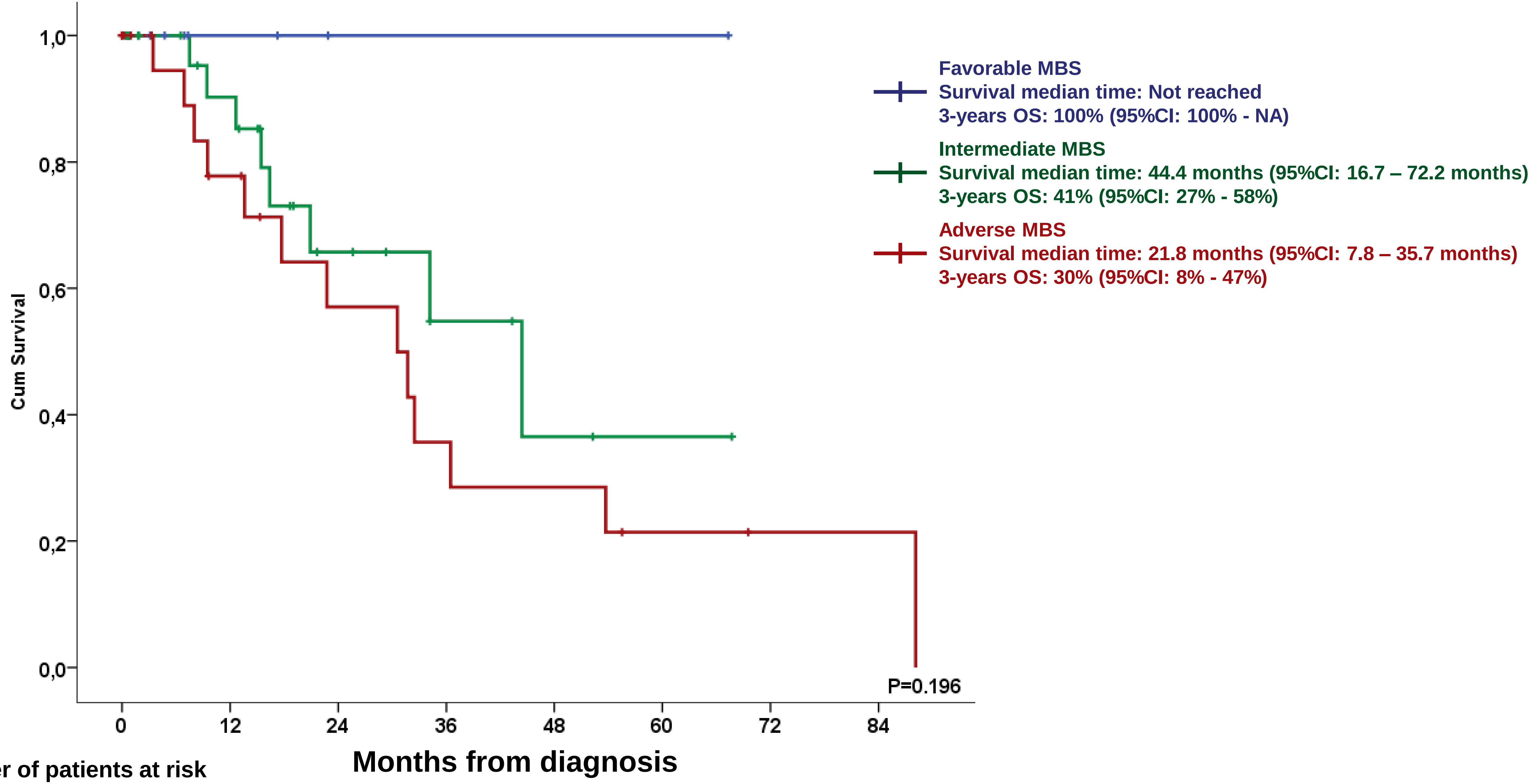

Number of patients at risk

Favourable	7	3	1	1	1	1	0	0
Intermediate	33	18	8	4	2	1	0	0
Adverse	23	13	8	5	4	2	1	1
